# The role and benefits of accessing primary care patient records during unscheduled care: a systematic review

**DOI:** 10.1186/s12911-017-0523-4

**Published:** 2017-09-22

**Authors:** Tom Bowden, Enrico Coiera

**Affiliations:** Centre for Health Informatics Australian Institute of Health Innovation, Macquarie University, Sydney, Australia

## Abstract

**Background:**

The purpose of this study was to assess the impact of accessing primary care records on unscheduled care. Unscheduled care is typically delivered in hospital Emergency Departments. Studies published to December 2014 reporting on primary care record access during unscheduled care were retrieved.

**Results:**

Twenty-two articles met inclusion criteria from a pool of 192. Many shared electronic health records (SEHRs) were large in scale, servicing many millions of patients. Reported utilization rates by clinicians was variable, with rates >20% amongst health management organizations but much lower in nation-scale systems. No study reported on clinical outcomes or patient safety, and no economic studies of SEHR access during unscheduled care were available. Design factors that may affect utilization included consent and access models, SEHR content, and system usability and reliability.

**Conclusions:**

Despite their size and expense, SEHRs designed to support unscheduled care have been poorly evaluated, and it is not possible to draw conclusions about any likely benefits associated with their use. Heterogeneity across the systems and the populations they serve make generalization about system design or performance difficult. None of the reviewed studies used a theoretical model to guide evaluation. Value of Information models may be a useful theoretical approach to design evaluation metrics, facilitating comparison across systems in future studies. Well-designed SEHRs should in principle be capable of improving the efficiency, quality and safety of unscheduled care, but at present the evidence for such benefits is weak, largely because it has not been sought.

## Background

One of the key justifications for developing Shared Electronic Health Records (SEHRs) is their potential to improve the quality and outcome of care for unanticipated or unscheduled events such as emergencies. In 1998, British Prime Minister Tony Blair famously stated that “If I live in Bradford and fall ill in Birmingham then I want the doctor treating me to have access to the information he needs to treat me” [1] as a justification for embarking on the £13 billion National Programme for Information Technology (NPfIT). Similar large-scale SEHR projects have been undertaken in many other countries including Canada, Australia and The United States of America.

Shared records come in many forms. Some are special-purpose ‘summary care records’ stored in centralized repositories [2]. Others, such as some Health Information Exchanges (HIEs), take a decentralized approach by creating a virtual health record that is assembled from working clinical record systems. Some SEHRs are government-owned and operated. Other nations take a less-direct “middle-out” approach, emphasizing the development of interoperability standards and encouraging the IT industry to work directly with the healthcare system [3, 4].

A surprising feature of most SEHR initiatives is that they have proceeded ahead of any significant body of research evidence for their likely costs and benefits. Since the early evaluations of the UK experience with SEHRs by Greenhalgh et al. between 2008 and 2012 [5–14], there has been a steady accumulation of evidence from post-hoc evaluations of other shared record projects. Parallel literature from the US has explored the benefits of Health Information Exchanges (HIEs), but the generic nature of these systems makes it hard to identify any specific impact on unscheduled care [15–17].

We undertook a systematic review of the published literature to summarize the evidence for costs and benefits of using electronic patient records created in primary care during unscheduled care. Unscheduled care is defined as any care that cannot reasonably be foreseen or planned in advance of contact with a health professional. Unscheduled care, by definition, is urgent with the need to take action at the time of contact with services [18].

## Methods

We undertook a PRISMA-compliant systematic review of studies to be included in the review, studies needed to meet the following criteria:

- The article was published in English;
- The article reported the use or impact of a SEHR on unscheduled care;
- The record system included primary care records;
- The study reported quantitative outcomes using randomized controlled trials, quasi-experimental studies, before-and-after studies, case-control studies, cohort- or case studies and cross-sectional studies.

Articles were searched for using Scopus, PubMed and Google Scholar with no date restriction and using the search string (“primary care” OR “general practice”) AND (“unscheduled care” OR “emergency care” OR “after-hours care”), AND ((“Health Information Exchange” OR HIE) OR (“Electronic Health Record” OR EHR)). Abstracts were excluded if they did not report study data. Study quality was assessed by examining study design, bias risk, study duration and population size.

**Fig. 1:**
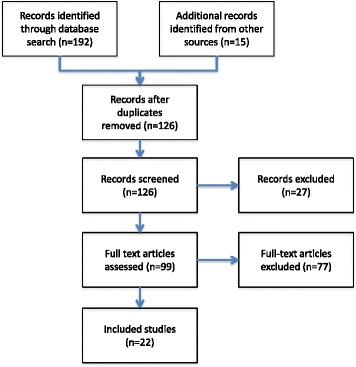
Systematic review flow diagram for articles reporting on the use of primary care records to support unscheduled care

Search identified 192 potential articles. An additional 15 articles were found by hand searching or citation following. After assessment against inclusion criteria, 22 studies remained (Fig. 1). Study data were extracted using a standardized template that covered system architecture, scale, level of uptake, and impacts on clinical outcomes or patient safety.

## Results

### Quality of studies

There was substantial heterogeneity in health system, patient population, technological approach and study methods across the 22 included studies, and pooled analysis was thus not possible (Table 1). There were no formal prospective controlled trials. As not every study reported against the same outcome measures, there is a risk that some studies were biased to report favourable rather than unfavourable outcomes.

**Table 1:** Summary of Included Articles

Paper	Study Design (Duration)	Scale	Uptake	Impact on Patient Safety	Impact on Clinical Care
*Great Britain*					
1. Greenhalgh et al. 2013 [5]	Retrospective comparative analysis	Four different shared records systems in each of: Scotland (5.1 million people), Northern Ireland (1.8 million people), England (51 million people) and Wales (3 million people).	230,000 monthly accesses of Scottish ECS (Emergency Care Summary). English (SCR) Summary Care Record temporarily halted.	Nil reported	Nil reported
*Scotland*					
2. Morris L, Brown C, Williamson M and Wyatt J. 2012 [26]	Survey	5.1 million people			Benefits claimed included more efficient assessment; reduced drug interactions; fewer adverse drug reactions; less duplicate prescribing; 34% of clinicians said the ECS had changed a clinical decision.
England					
3. Ayatollahi H, Bath P A and Goodacre S. 2009 [38]	Qualitative study/survey (2 months)	Hospital with 1100 beds and 5500 staff servicing population of 530,000	Mostly using paper records	Nil reported	Occasional clinical benefits from access to patient information in ED noted. Patient confidence in the confidentiality of information is paramount.
4. Greenhalgh et al. 2008 [6]	Multi-site, mixed-method case study	51 million people (1 year)	System was discontinued	The study found that there was no direct evidence of improved clinical safety apart from a ‘rare but important positive impact on preventing medication errors.’	The study identified that having pre-existing records delivered a rare but important ‘positive impact on preventing medication errors’.
5. Greenhalgh et al. 2008 [9]	Semi-structured interviews and focus groups	51 million people	The project was halted and is still in the process of being restarted.	Nil Reported	Nil Reported. This study did not study actual usage of the system. It focused on the patients’ attitudes toward use of it.
6. Greenhalgh et al. May 2010 [8]	Retrospective observational study, interviews and ethnographic field observation.	16 Primary Care trust regions with a total population of 29.8 million (1 year).	By 2010, 1.5 million such records had been created. In participating primary care out-of-hours and walk-in centers, an SCR was accessed in 4% of all encounters and in 21% of encounters where one was available.	Nil observed, though risks seen from patient records being incomplete or inaccurate.	Rare but important positive impact noted where system helped in preventing medication errors.
U.S.					
7. O’Malley A S, Samuel D, Bond A M, and Carrier E. 2012 [39]	Interviews	16 US States (6 months)	Very limited - only 29% of general practices make after hours care arrangements.	Nil Reported	Most of the benefits determined were based on cost containment and in some cases revenue generation and in-network referral retention.
8. Vest J R, Gamm L D, Ohsfeldt R L, Zhao H and Jasperson J. 2012 [35]	Retrospective observational study	Integrated care collaboration of central Texas. Population not stated (3 years).	HIE was used for up to 21.1% of encounters.	Nil Reported	Nil Reported
9. Frisse M E, Johnson K B, Nian H, Davison C, Gadd C S, Unertl K M, Turri P A and Chen Q. 2011 [33]	Retrospective matched cohort analysis	Memphis Tennessee, 1.2 million people (2 years)	HIE data was accessed in 6.8% of all visits.	Use of HIE reduced hospital admission rates.	Use of HIE improved management of patients with long-term conditions resulting in $1.07 million annual savings.
10. Hripcsak G, Sengupta S, Wilcox A and Green R A. 2006 [40]	Retrospective matched cohort analysis	2.5 million patients (7 months)	Used for 5–30% of patient encounters.	There were information gaps for one third of ED patients. In one third of those cases, getting that missing information was important.	Closing gaps in the information provided to EDs results in significantly increased efficiencies in care; a reduction of both redundant testing and treatment delays, enabling scare resources to be redeployed for care of other patients.
11. Shapiro J S, Kannry J, Kushniruk A W and Kuperman G. 2007 [41]	Survey	Survey of 216 emergency physicians across 12 New York hospitals	The emergency physicians surveyed believe that reliable availability of information will lead to significant usage.	Nil observed; Please note: This was a survey of opinions and attitudes about future HIE usage rather than a survey of actual HIE usage.	Nil observed, Please note: This was a survey of opinions and attitudes about future HIE usage rather than a survey of actual HIE usage.
12. Vest J R and Jasperson S 2011 [42]	Retrospective observational study	The medically indigent population of central Texas (3.5 years)	105,705 unique users’ sessions is a significant number of observations	Nil Reported	Nil Reported
13. Vest J R, Kern L M, Silver M D and Kaushal R. 2014 [43]	Retrospective observational study	800,000 patients in Rochester, New York (2 years)	6800 records were analyzed.	A significant decline in readmission indicates that the system has value in reducing adverse outcomes.	A significant decline in readmission (57%) indicated that the system has value in improving patient care.
14. Finnell J T, Overhage J M. 2010 [19]	Track log file analysis	1.6 million people in Indiana (6 months)	HIE information sought in 16% of ED admissions.	Nil reported	Nil reported, however the majority of clinicians viewed the availability of information as beneficial.
15. Johnson K B, Unertl K M, Chen Q, Lorenzi N M, Nian H, Bailey J, Frisse M. 2011 [28]	Mixed-method analysis, observation and interviews	1.7 million patients in Memphis (6 months)	Used for 7% of all patients and in 16% of repeat visits.	Detected public health risks on a small number of patient visits (0.8%).	Reduced the time taken to see patients, reduced the need for repeat testing and improved clinicians’ understanding of patients’ overall conditions.
16. Yaraghi N. 2015 [27]	Retrospective Observational study	Unstated (6 months)	737 ED visits	Nil reported	Significant reductions in laboratory tests (52%) and use of radiology services (36%) with the resultant ability to redeploy resources to other aspects of care.
Netherlands					
17. Dumay A C M and Haaker T I. 2010 [21]	Interviews	The Twente Region (population 620,000)	The electronic locum report (ELR) system is well used in Twente but efforts to scale it up and expand it across the Netherlands have failed.	Nil Reported	Nil reported. This study did not assess the value of the ELR system as an aid to improving clinical care.
18. Woudstra D P J. 2013 [20]	Interviews	Holland (population 16.8 million).	Only 23.5% of Dutch population have given permission for their electronic health records to be shared.	Nil reported. This study did not look at improvements to patient safety, nor did it measure any impact on the quality of patient care that could be achieved via use of the ELR.	Nil reported. This study did not look at improvements to patient safety, nor did it measure any impact on the quality of patient care that could be achieved via use of the ELR.
Israel					
19. Ben-Assuli O, Shabtai I and Leshno M. 2013 [24]	Track log file analysis	Seven main Israeli hospitals; 3.8 million patients (HMO) (3 years)	Medical history viewed in 16.2% of referrals.	Access to records improves admission decisions. Good admission decisions have a significant impact on patient safety. Although many ED physicians believe that the majority of their patients would benefit from longitudinal health information, they attempt to obtain such data less than 10% of the time.	An improved admission decision positively impacts a patient’s clinical care and improves the medical facility’s ability to manage its resources in a manner that enables optimal care delivery. Access to internal records resulted in a 22.9% reduction in single day admissions. The availability of a patient’s long term health records including information about medications, diagnoses, recent procedures, and recent laboratory tests is critical to forming an appropriate plan of care.
20. Ben-Assuli O, Leshno M and Shabtai I. 2012 [22]	Track log-file analysis	Seven main Israeli hospitals 3.8 million patients (HMO) (3 years)	Medical history viewed in 16.2% of referrals.	Physicians used medical records more when under pressure. Emergency physicians may admit more patients unnecessarily when under pressure, if they do not have time to get access to the information they need.	Records found to be more useful in complex cases.
21. Ben-Assuli O, Shabtai I and Leshno M. April 2013 [25]	Track log file analysis	Seven main Israeli hospitals; 3.8 million patients (HMO) (3 years)	External medical history viewed in 4.3% of cases. Internal medical history was viewed in 26.9% of cases.	Reduced the number of emergency readmissions within 7 days: confirms a clear improvement in patient safety.	Improving admission decisions positively impacts care, freeing up resources to better focus care on where it is needed.
22. Ben-Assuli O, Shabtai I, Leshno M and Hill S. 2014 [23]	Track log file analysis.	Seven main Israeli hospitals 3.8 million patients. (3 years)	Medical history viewed in 24% of all referrals.	Better admission decisions improve patient safety. Availability of blood pressure results increased the likelihood of admitting a patient by 70.6%. Availability of community records increased the likelihood of admitting CP patients by 29.2%.	Better admission decisions improve quality of care. Better decisions are enabled by a more comprehensive patient view.

### Scale of records

Many of the reported SEHRs were large in scale. The four countries of the United Kingdom covered large populations where the majority of people had a shared record (Table 2): England (80% of 51 million people), Scotland (99% of 5.1 million), Wales (65% of 3 million), and Northern Ireland (99% of 1.8 million) [5]. Other studies reported SEHRs operating over regions, and some of these were large in scale. In the US the largest regional system reported was in Indiana, covering 10.2 million patient records [19]. In the Twente region of Holland, 49% of the 620,000 population had records [20, 21] and Israeli health maintenance organizations had records for a population of 3.8 million people [22–25].

**Table 2:** Scale and utilization of shared electronic health records (SEHR) in unscheduled care (HMO = health maintenance organization; ED = Emergency Department)

Setting	Population Size	Patients with a record	Patients opted-out % (n)	General Practices connected to SEHR % (n)	SEHR access
England [5–9, 38]	51 million	80%	1.4% (714,000)	Not Reported	3.2 accesses per GP per month (82,000 total)
Scotland [5, 26]	5.1 million	99%	0.03% (2000)	100% (970)	46 accesses per GP per month (230,000 total)
Wales [5]	3 million	65%	Not Reported	65% (290)	Not Reported
Northern Ireland [5]	1.8 million	99%	Not Reported	100% (354)	Not Reported
United States of America					
Integrated Care Collaboration of Central Texas [35]	Not Reported	6393 patients included in the study	1.5% (96)	Two urban community health centers – both participated	21% of all encounters
The Midsouth eHealth Alliance of Memphis Tennessee representing 12 major hospitals [33]	1.7 million patients	1.7 million	1–3% (study conducted across multiple sites)	Not Reported	6.8% of ED encounters
New York-Presbyterian Hospital/ Columbia University Medical Center [40]	2.5 million patients	2.5 million	Nil	Not Reported	20–50%
Indiana Network for Primary Care [19]	Not Reported	10.2 million records	Not Reported	Not Reported	26% of all ED contacts
HealtheLink the regional health information exchange of Western New York [27]	Not Reported	737 patients studied	5.3% (39 patients)	Not Reported	100% of records were accessed for patients in the study cohort
Rochester New York area [43]	1.14 million	800,000	Nil	Not Reported	14.21% of ED visits
Holland (Twente region) [20, 21]	620,000	49%	51% of people did not opt in	95%	Not Reported
Israel (HMOs) [22–25]	3.8 million	100%	Not Reported	100%	23.7% of all ED encounters

Few studies provided data on the percentage of primary care services connected to the SEHR. Those that did reported high connection rates: Wales (65%), Holland (95%), Scotland (100%) and Israel (100%).

### Utilization of records in unscheduled care

The rate at which SEHRs were accessed by clinicians was less well reported (Table 2). Amongst the large-scale SEHRS, reported rates of record access per patient encounter varied significantly. High rates were reported for Israel (23.7%), New York/Columbia (20–50%), the Indiana Network (26%) and Rochester (14%). Lower rates were reported in Memphis (6.8%). In the UK, utilization was reported as the number of accesses per primary care physician per month: England (3.2), Scotland (46).

### Improved quality and safety of care

None of the studies measured the impact of the SEHR on the quality or safety of patient care. It is therefore not possible to report on whether the use of an SEHR is of any clinical or health service benefit in quantitative terms. Several studies did provide qualitative evidence of benefit.

A review of the Scottish system noted that “it is difficult to prove specific clinical benefits”, as a randomized trial was considered unethical [26]. The Scottish review reported that 36% of clinicians identified instances in which SEHR medicines data did not match patient reports, signalling either deficiencies in the SEHR or patient supplied data. Over 81% of Scottish clinicians surveyed rated the system as helpful or very helpful and reported that it had changed their management in 20% of accesses. Respondents stated that the SEHR was particularly helpful if patients were confused or receiving multiple medications. Experienced clinicians working in Accident and Emergency Departments, who used the system infrequently, saw benefits for patients with multiple co-morbidities or medications. Examples provided included patients presenting who were unable to communicate because of strokes or dementia, as well as patients with allergies. In Israel, access to an SEHR was reported to improve admission planning for cardiac patients via a reduction in the number of avoidable single-day admissions. Overall the number of single day admissions dropped by 17.3% [24].

### Economic impact

No system-wide economic benefit analyses were reported. A US study reported a 52% reduction in laboratory tests and a 36% reduction in radiology examinations ordered per patient at a single emergency department as a consequence of accessing patient data from a health information exchange [27]. This study did not calculate financial savings, nor assess whether reductions in service utilization were appropriate i.e. whether some of the tests were inappropriately not ordered.

### Record content

The content of records in SEHRs varied considerably. Some provided full access to clinical records. In the US, Health Maintenance Organizations (HMOs) provided access to all the EHR data held in the HMO’s systems [28]. Israeli HMOs provided complete access to GP records [25]. In Wales, an extract from the GP record was provided, with certain sensitive fields obscured [5].

In contrast, the Scottish and Northern Irish systems contained only demographic information, current- and discontinued medications, and adverse reactions [5]. In Scotland, an even smaller subset of patient data included currently prescribed medications and adverse reactions [5]. The English Summary Care Record (SCR) contained core information such as details of medications (including long-term-, acute- and discontinued medications), allergies, and adverse reactions. The SCR could also hold additional information e.g. management of long-term conditions, end of life or mental health care plans [5, 8]. In Holland, the electronic locum record provided a summary of information about the patient which included significant health problems, the most recent GP visit records, current medications and allergies [21].

### Patient consent and clinician access controls

Studies reported three broad approaches to obtaining patient consent to share records [4]:

1. “Opt Out” systems, where patients are informed that unless they request otherwise, their records will be automatically available to be shared;
2. “Opt in” systems where patients are asked to confirm that they are happy for their records to be made available;
3. Hybrid models that combined an implied consent for records to be created and an explicit consent to view.

The rate at which patients opt out of an SEHR may affect the utilization rates and benefits of the system. The fewer patients enrolled in a system, the lower the probability that a clinician will find a record when they query the system. The opt-out rates reported were low: Scotland (0.03%), England (1.3%), Texas (1.5%) and Memphis (1–3%). In the US consent models and privacy legislation varied from state to state. Patients belonging to an HMO are deemed to have opted in by subscribing to the HMO.

All of the UK SEHRs employed hybrid consent models including a ‘break the glass’ mechanism to access records if the patient is too ill or unable to consent [5]. In Wales a clinician has unrestricted access to records but all usage is subject to auditing [5]. In England the SCR began with a similarly weak consent model with records uploaded and viewable by any healthcare provider who claimed a legitimate relationship with a patient. GP access requires a SmartCard with Role-Based Access Controls (RBAC) and all accesses are auditable [5]. In Wales there was no role-based access control but periodic audits were undertaken [5].

In Holland, the Electronic Locum Record (ELR) in the Twente region began on an “opt out” basis but changed to opt-in following changes to national law. By June 2014, 3.95 million Dutch people (23%) had opted-in nationally compared to 49% in Twente.

### System usability and reliability

In England, the US and The Netherlands, unreliability of the system in use, or interruption to access because of problems with a computer network, were seen as key reasons for clinicians not accessing the SEHR [8, 19, 20]. In the US, not having data integrated within ED systems reportedly reduced usage, although no data was provided. Making it easy for ED clinicians to use an SEHR by reducing the number of keystrokes; removing the need to toggle between user interfaces; and reliably presenting data within the ED system were all seen as ways in which usage could be encouraged [21].

### System design

The approaches taken with SEHR operation were diverse. In Scotland, Wales and in Northern Ireland, GP record updates are automatically uploaded twice daily to a centralized database [26]. In England, GP records were updated every time a GP logged onto the secure national network [5]. Across the UK, after-hours services automatically query the central patient database each time a consenting patient makes information available to the treating clinician [26].

In the US, a number of different technical approaches are used by HIEs to combine data from multiple sources into a single accessible electronic health record. These range from a federated architecture where patient information is held in multiple locations and combined when queried, to centralized approaches where a single patient record is used [26, 29].

### Implementation approach

The English NPfIT was an example of top-down program delivery. NPfIT encountered many technical, procurement and clinical end-user challenges, resulting in its suspension in 2011 following reported expenditure of £1.5 billion on the Summary Care Record (SCR) alone. The current English strategy has much stronger end-user engagement and local involvement in system procurement and implementation. All primary care trusts had at least one local champion, usually an enthusiastic general practitioner or senior nurse. Grass-roots support for the SCR also came from GPs who worked in After Hours services [8].

The Scottish system was developed by general practitioners, who were looking for a solution that would enable them to deliver after-hours care. There was extensive consultation within the medical community and the general public. Each region was implemented using local champions [26]. Introduction of the SEHR in Northern Ireland followed the Scottish approach [5]. In Wales, the introduction was part of a larger programme of health IT reform, including implementation of a national patient portal and clinical referrals gateway [5].

In Holland, lack of user training was considered to be a major barrier to uptake. In the Twente region, groups of medical practices helped one another to implement a bottom-up ‘home-grown’ system. Detailing how it would work, promoting use and instructing users were viewed as essential [21]. In Israel, the systems were implemented “top-down” by the country’s largest Healthcare Maintenance Organizations, responsible for delivery of a significant proportion of Israel’s healthcare [25].

In the US, most Health Information Exchanges (HIEs) have been implemented in a top-down manner, to comply with federal requirements. Most HIEs are organized on a not-for-profit basis and many are financially challenged, with a few prominent exceptions [30].

## Discussion

The studies in this review included many large-scale national and regional SEHRs, typically covering millions of individuals, and capable of supporting unscheduled care. A striking feature is the near complete absence of data regarding system impacts on the quality and safety of unscheduled care, or economic outcomes. Some evidence points to improved admission decisions [22, 24, 25] and reduction in unnecessary radiology and pathology procedures [27], but the significant variation in SEHR context and system design makes it hard to generalize these results to other settings. The situation is similar in the literature for scheduled care. In the US, most regional health information exchanges struggle with low provider engagement, and it is still not clear how information exchange affects the cost and quality of healthcare services [30].

One would expect that the huge investments required to design, build and implement such systems would be based on strong prior evidence, and would have triggered evaluation of system benefits post-implementation. Taken as a whole this suggests that, at least for this globally-significant class of system, the drivers for system design, development and operation are not yet evidence-based. This is underscored by the absence of peer-reviewed studies for many long-running national SEHRs such as Australia’s My Health Record system, Singapore’s National Electronic Health Record (NEHR) system, Hong Kong’s Electronic Health Record Sharing System (eHRSS) and Regional Health Information Exchange Organisations (RHIOs) in the US.

This review has been limited by the low number, and relatively poor quality, of published studies. No studies were prospective trials. Each study reported different systems in different contexts, and this heterogeneity makes generalizing results challenging. The review did not include government or organizational reports that were not peer-reviewed.

### The role of theory in evaluating the impact of electronic records

None of the papers reviewed took advantage of a theoretical framing to guide evaluation. As a consequence, little rationale was provided for the selection of one outcome measure over another. Yet without clear causal mechanisms for the changes one might expect from a SEHR, it is difficult to design studies that clearly separate any changes due to an SEHR from other events in a health system. It is also difficult to compare the different outcomes found in studies, given that systems and contexts varied so much.

**Fig. 2:**
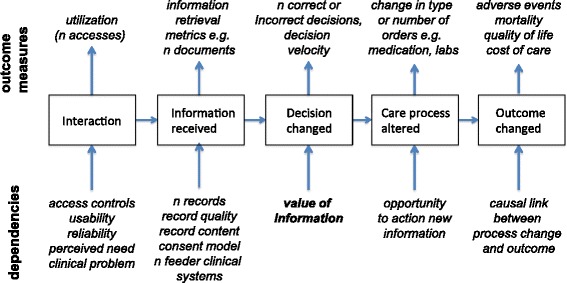
The information value chain provides a simple causal model connecting record system use and clinical outcomes. Each step is characterized by different measures, and is dependent on different elements of shared record system design and use (adapted from Coiera, 2015, Chapter 11)

The *Value of information* (VOI) models provide one such theoretical foundation [31]. Health records can only have impacts on care when the information they provide to decision-makers triggers a change in decision with the potential for a higher-value outcome. *Information value chains* can be constructed using this VOI [32]. For health records the value chain begins with clinicians interacting with the record system, then retrieving information, making a decision and initiating processes such as ordering a test or medication (Fig. 2). Outcome changes follow. Transitioning from one stage to the next is dependent on the previous stage and is not guaranteed as the probability of progression will vary with context and system.

For any formal evaluation of a SEHR, value chain analysis suggests that measurements need to be taken at multiple points in the decision-making process, and that variations in outcome at any stage can only be understood by modelling earlier upstream events. Thus, failure to demonstrate clinical outcome changes following the implementation of an SEHR might arise because of problems with events early in the chain e.g. record quality. Alternately, a lack of impact on outcomes may be unrelated to the SEHR (for example organizational challenges may prevent important information from the SEHR being translated into process changes).

### Utilization of records for unscheduled care

In the few cases where electronic record utilization rates were reported, there was considerable variation (Table 2). Relatively high rates were reported in several US studies (often rising above 20% of encounters). In contrast there were relatively low rates in England and Scotland with between 3 and 46 accesses per clinician per month. In a 2011 study of the Memphis Metropolitan area, 12 ED sites and nine ambulatory care clinics found that users accessed records for 6.8% of encounters, with higher rates for repeat visitors, patients with co-morbidities, patients known to have pre-existing data and at sites where access by both nurses and doctors was allowed. Discharge summaries and test reports were the most frequently accessed items [28, 33].

Access rates are likely to be dependent on a number of factors. The complexity of access controls, as well as system usability and reliability, are all likely to influence a clinician’s decision to use an SEHR. If the expectation is that records will not be available, or that information is unlikely to be useful, then the perceived need that drives access will also be low [5, 26, 28]. The lower UK access rates may be partially explained by the need to toggle between the SEHR and clinical systems, and the record being only a small subset of the full record.

Higher utilization studies typically came from HMOs, where accessing the SEHR was part of the normal EHR workflow, and the clinical data available came from the entire health record. Few studies reported on the number of health services providing data into the SEHR. Those that did were mainly in the UK, and reported very high levels of connection, probably reflecting that these health systems were largely public rather than private. In contrast, despite extensive investment in Health Information Exchange (HIE) technology in the US, only 14% of US physicians reported sharing data with providers outside their organization [34].

In Israel, clinicians reported higher SEHR utilization rates when the ED was under pressure [22]. In the Indiana Network for Primary Care, physicians identified specific patient groups for whom SEHR information was of greater value in unscheduled care. These groups included patients who could not communicate their health history (for example those who were unconscious, uncooperative or intoxicated) and elderly patients with existing co-morbidities [19]. Patients with a low perceived need for additional information included those with trauma or able to clearly express their history [19, 28].

### Implications for policy and practice

There is urgent need for better evaluation studies of safety, quality and outcomes. The level of evaluation reported here is incommensurate with the expense and effort involved in creating SEHRs. With record systems typically servicing millions of patients, it would seem both prudent to demonstrate value for money in making this investment, as well as to demonstrate clear clinical benefit. With health budgets constrained everywhere, expenditure in one area means reduction of resources elsewhere. Given that SEHRs appear to increasingly be politically sensitive and controversial systems, formal and well-designed evaluations should be an essential project element whenever planning, implementing, or operating an SEHR. An SEHR is not a singular health service intervention, but is a bundle of different technical and operational elements, along with an implementation strategy. When creating an SEHR one would hope that the existing evidence base could help in customizing this bundle to best suit the needs of a particular health service. At present such an evidence base does not appear to exist.

When implementing a shared record system, maximizing utilization should be a focus. Whilst utilization of an SEHR is no guarantee that it will deliver the outcomes and cost-benefits, nothing is likely to be delivered in the absence of healthy utilization rates. Barriers to utilization include poor system usability, reliability [19, 21] and utility. If systems are hard to use, erratically available, do not fit into the pre-existing workflow [33, 35], or deliver no perceived benefit, then clinicians are unlikely to use them. This suggests that SEHR projects should focus strongly on clinical needs and work practices, to minimize the barriers to SEHR access and maximize the clinical value of information retrieved.

The selection of record content is likely to shape utilization rates. In unscheduled care, one would expect a minimum clinical summary to cover data that would have an immediate impact on the care delivered. Allergies, current medication and diagnoses are likely to be useful in triage and immediate treatment decisions, whereas access to the full record will probably deliver higher value at a later stage in a patient’s workup. Utilization rates appeared higher when a full record is available, typically in US HMOs [19, 27]. It is not clear, however, whether these rates are inflated by episodes of scheduled care.

Privacy and consent models that do not meet the expectations of the community can lead to program failure. Consent models can be hard to evaluate and difficult to choose from, but their implementation is vital to the success of SEHRs. Whilst privacy and consent concerns are commonly expressed by consumer organisations, typically there is also strong support for SEHRs amongst those with chronic illness, or other reasons to regularly engage with the health system [36]. The desire by consumers to explicitly decide if their records are placed into an SEHR, and the controls they wish over record access, will be influenced by events in the public domain. The increasing prevalence of privacy and security breaches involving medical records is likely to diminish trust in system security. Failure to disclose which data are collected, and who is authorized to use them, can have a major impact. Denmark’s DAMD (The Danish General Practice Database) system, for example, was authorized to collect a small subset of important data and records of 4.9 million patients. However, in November 2014 it was discovered that DAMD was holding a much larger set of patient data than authorized, forcing immediate suspension of the system. A major challenge with privacy is that consent models for data sharing such as opt-in or opt-out are often conflated with access control strategies. For example consumer opt-in is seen by some as a better privacy model because there has been an informed decision to share data, whereas opt-out might end up seeing data shared without explicit patient understanding or consent [37]. Yet one can have an opt-in system which gives clinicians access to all records with minimal auditing – a very poor approach to privacy. Alternatively an opt-out system that placed stringent gatekeeper demands on clinicians to prove who they are, that they have the right to access a document, and that audits any document access is likely to be a very secure system.

Middle-out programs appear more likely to engage stakeholders and ultimately succeed. Large-scale health IT programs have the option of being driven top-down by governments and large organisations, bottom-up driven by the end user community, or a blended ‘middle-out’ approach [37]. The evidence reported here seems to suggest that, at least in terms of system implementation and user acceptance, SEHR projects fare best when they are collaborative “middle out” ventures. There need to be clear roles for different authorities and users. Distribution of ownership and input into design and implementation decisions seems to be effective.

### Limitations of this review

We can only cautiously generalize the lessons from the studies reported here, because of the significant variation in record systems and context of use, and the often weak nature of study designs. This heterogeneity across system design, implementation strategy and population also precluded any meta-analyses.

The search strategy for this review would have missed any published system evaluations that did not use one of the keywords “unscheduled care”, “emergency care” or “after-hours care”. Equally many systems that are in operation do not have published evaluations in the scientific literature at this point in time. Some EHR systems such as HIEs may have provided functions that support unscheduled care, but the generic nature of evaluations for these system made it hard to identify specific impacts on unscheduled care.

As a result there is always a risk of bias in the sample of systems reported on, and it is possible that data from these unreported systems is somehow different to those reported on. For example, there may be disincentive to publish evaluations of systems where performance has been poor, and their inclusion may have painted a different picture.

## Conclusions

Shared electronic records, if well designed and appropriately targeted to meet specific and high value informational needs, should in principle improve the quality, safety and effectiveness of clinical care. At present however, the evidence for such benefits is weak, largely because it has not been sought. Given the scale and cost of such systems, this absence of evidence is both surprising and concerning.

It is also the case that there has been little clarity in connecting the informational needs which arise during unscheduled care with system design and scale. The lack of theoretical models to underpin SEHR design and evaluation means that some of the systems surveyed may not have been fit for purpose [33], but rather were generic technology driven endeavours. Seeing the SEHR as part of an information value chain emphasizes that information delivery must be connected to decision making, for example through decision support systems, to deliver the most value.

## Abbreviations

ED: Emergency Department
EHR: Electronic Health Record
HIE: Health Information Exchange
NPFIT: The English National Health Service’s National Programme for Health Information Technology (now concluded)
SEHR: Shared electronic health record
